# In Vitro Superparamagnetic Hyperthermia Employing Magnetite Gamma-Cyclodextrin Nanobioconjugates for Human Squamous Skin Carcinoma Therapy

**DOI:** 10.3390/ijms25158380

**Published:** 2024-07-31

**Authors:** Isabela-Simona Caizer-Gaitan, Claudia-Geanina Watz, Costica Caizer, Cristina-Adriana Dehelean, Tiberiu Bratu, Zorin Crainiceanu, Adina Coroaba, Mariana Pinteala, Codruta-Marinela Soica

**Affiliations:** 1 Department of Plastic and Reconstructive Surgery, Faculty of Medicine, “Victor Babes” University of Medicine and Pharmacy of Timisoara, 300041 Timisoara, Romania; isabela.caizer@umft.ro (I.-S.C.-G.); tiberiu.bratu@umft.ro (T.B.); crainiceanu.zorin@umft.ro (Z.C.); 2 Department of Clinical Practical Skills, Faculty of Medicine, “Victor Babes” University of Medicine and Pharmacy of Timisoara, 300041 Timisoara, Romania; 3 Advanced Cardiology and Hemostaseology Research Center, “Victor Babes” University of Medicine and Pharmacy of Timisoara, 300041 Timisoara, Romania; 4 Department of Pharmaceutical Physics, Faculty of Pharmacy, “Victor Babes” University of Medicine and Pharmacy of Timisoara, 300041 Timisoara, Romania; farcas.claudia@umft.ro; 5 Research Centre for Pharmaco-Toxicological Evaluation, “Victor Babes” University of Medicine and Pharmacy of Timisoara, 300041 Timisoara, Romania; cadehelean@umft.ro (C.-A.D.); codrutasoica@umft.ro (C.-M.S.); 6 Department of Physics, Faculty of Physics, West University of Timisoara, 300223 Timisoara, Romania; 7 Department of Toxicology, Drug Industry, Management and Legislation, Faculty of Pharmacy, “Victor Babes” University of Medicine and Pharmacy Timisoara, 300041 Timisoara, Romania; 8 Center of Advanced Research in Bionanoconjugates and Biopolymers, “Petru Poni” Institute of Macromolecular Chemistry of Iasi, Romanian Academy, 700487 Iasi, Romania; adina.coroaba@icmpp.ro (A.C.); pinteala@icmpp.ro (M.P.); 9 Department of Pharmacology-Pharmacotherapy, Faculty of Pharmacy, “Victor Babes” University of Medicine and Pharmacy, 300041 Timisoara, Romania

**Keywords:** A431-human epidermoid squamous carcinoma, in vitro, alternative therapy, superparamagnetic hyperthermia, magnetite gamma-cyclodextrins, nanobioconjugates

## Abstract

In vitro alternative therapy of human epidermoid squamous carcinoma (A431) by superparamagnetic hyperthermia (SPMHT) using Fe_3_O_4_ (magnetite) superparamagnetic nanoparticles (SPIONs) with an average diameter of 15.8 nm, bioconjugated with hydroxypropyl gamma-cyclodextrins (HP-γ-CDs) by means of polyacrylic acid (PAA) biopolymer, is presented in this paper. The therapy was carried out at a temperature of 43 °C for 30 min using the concentrations of Fe_3_O_4_ ferrimagnetic nanoparticles from nanobioconjugates of 1, 5, and 10 mg/mL nanoparticles in cell suspension, which were previously found by us to be non-toxic for healthy cells (cell viabilities close to 100%), according to ISO standards (cell viability must be greater than 70%). The temperature for the in vitro therapy was obtained by the safe application (without exceeding the biological limit and cellular damage) of an alternating magnetic field with a frequency of 312.4 kHz and amplitudes of 168, 208, and 370 G, depending on the concentration of the magnetic nanoparticles. The optimal concentration of magnetic nanoparticles in suspension was found experimentally. The results obtained after the treatment show its high effectiveness in destroying the A431 tumor cells, up to 83%, with the possibility of increasing even more, which demonstrates the viability of the SPMHT method with Fe_3_O_4_-PAA–(HP-γ-CDs) nanobioconjugates for human squamous cancer therapy.

## 1. Introduction

Magnetic hyperthermia (MHT) and recently superparamagnetic hyperthermia (SPMHT) are very promising alternative methods for future cancer therapy without radiation and cytostatics, as is the case with conventional therapies, that can be more effective in destroying tumor cells and without toxicity on the living organism [1,2,3,4,5,6,7,8]. Magnetic hyperthermia uses an external harmonic alternating magnetic field of the order of hundreds of kHz and biocompatible magnetic nanostructures/nanoparticles [9,10] that are introduced into the tumor by different techniques employing predetermined concentrations. Thus, under the action of the alternating magnetic field, the magnetic nanoparticles heat up, causing the temperature of the tumor cells to rise in the range of 42–45 °C, leading to their irreversible destruction via apoptosis [3,11,12]. Therefore, magnetic hyperthermia is based on the natural thermal effect of heating tumor cells without the use of radiation and drugs as in the case of classical therapies (radio- and chemotherapy) which have a high degree of toxicity on the living organism.

Previous studies have shown that magnetic hyperthermia with small nanoparticles generally smaller than 15–20 nm (depending on the nature of the material), which have a superparamagnetic behavior in a magnetic field [9,13] known as superparamagnetic hyperthermia (SPMHT) [2], is more efficient from the point of view of the specific loss power (SLP) by the magnetic nanoparticles and the heating temperature than the classic magnetic hyperthermia that uses larger magnetic nanoparticles or nanostructures (generally >20–25 nm) [14]. Moreover, large magnetic nanoparticles or nanostructures give high levels of cellular toxicity compared to small magnetic nanoparticles, where toxicity is greatly reduced or even eliminated in the case of highly biocompatible nanoparticles [10].

The most-used nanoparticles for magnetic hyperthermia are the ferrimagnetic ones of iron oxides, represented by magnetite (Fe_3_O_4_) or γ-Fe_2_O_3_, which manifest the most suitable magnetic properties for magnetic hyperthermia and also present reduced cellular toxicity at their low concentrations, even in the absence of a biocompatible organic layer on their surface. However, studies are also being performed to develop other nanoparticles and magnetic nanocomposites [15] that lead to higher efficacy and reduced toxicity (or no toxicity) for cancer therapy through magnetic hyperthermia [5,6,16,17,18] and other current alternative methods (photothermal therapy (PTT) and photodynamic therapy (PDT)) [19], or in magnetic resonance imaging (MRI) [20].

In addition, in superparamagnetic hyperthermia, the magnetic field used has small amplitudes, from kA/m to several tens of kA/m (max. 30–40 kA/m), compared to the one used in magnetic hyperthermia, where it must have high or very high values (>100–200 kA/m, depending on the material) to obtain the effect that is desired, namely, the magnetic hysteresis. In this case, the loss power of the magnetic nanoparticles is proportional to the area of the hysteresis loop obtained as a result of their magnetization in the external magnetic field. A high-amplitude field which exceeds the maximum admissible biological limit (H × f = 5·10^9^ Am^−1^ Hz, H is field amplitude and f is frequency of magnetic field) [21] becomes toxic for healthy cells/tissues, a fact that is undesirable in magnetic hyperthermia therapy.

Therefore, considering the above aspects, in order to increase the efficiency and reduce the toxicity (or even eliminate it) of cancer therapy, it is necessary to respect the following aspects: employing superparamagnetic hyperthermia (SPMHT) with small magnetic nanoparticles (superparamagnetic), using only highly biocompatible nanoparticles, and requiring low magnetic fields that do not exceed the admissible biological limit.

Considering these, we previously proposed [22], based on our studies [2] and others [23,24], the use of superparamagnetic hyperthermia with biocompatible core-shell Fe_3_O_4_-PAA–(HP-γ-CDs) nanobioconjugates for effective and non-toxic cancer therapy, where Fe_3_O_4_ iron oxide nanoparticles (magnetite core) are superparamagnetic (SPIONs) and coated with hydroxypropyl gamma-cyclodextrins (HP-γ-CDs) by means of polyacrylic acidic (PAA) biopolymer (shell). The HP-γ-CDs nanobiostructure was found by our research group to be the most stable of the α-, β-, and even γ-CDs (CDs: cyclodextrins) and, at the same time, to be perfectly biocompatible even up to high concentrations of 10 mg/mL (for nanoparticles in PBS), with the cell viability experimentally determined to be close to 100% [22]. This is due to the well-known fact that cyclodextrins are biocompatible natural oligosaccharides, which are currently used in pharmaceuticals, foods, and cosmetics [25]. Apart from the excellent biocompatibility of HP-γ-CDs, we selected this nanobiostructure to cover the SPIONs of magnetite considering also the low thickness of the organic layer that is formed on the surface of the magnetic nanoparticles. Thus, it allows an increase in the concentration of SPIONs in the dispersion, with a beneficial effect on the specific loss power (SLP) in magnetic hyperthermia and, implicitly, the heating temperature of the magnetic nanoparticles, compared to the case of large biostructures [26], where the concentration of magnetic nanoparticles is limited. Moreover, by covering the SPIONs, the magnetic interactions between the nanoparticles are also eliminated, which would otherwise lead to the formation of very large agglomerates of nanoparticles with undesirable effects, reducing the efficiency and increasing the cell toxicity in superparamagnetic hyperthermia. In the case of magnetic nanoparticles covered with large biostructures, such as, e.g., liposomes, which were used much earlier in magnetic hyperthermia [8,27], the efficiency of this therapy can decrease dramatically due to the reduced concentration of magnetic nanoparticles in the injectable suspension.

The novelty of this study consists in the use of nanobioconjugates of Fe_3_O_4_-PAA–(HP-γ-CDs) (magnetic nanoparticles of Fe_3_O_4_ bioconjugated with HP-γ-CDs by means of polyacrylic acid (PAA) biopolymer) for the first time in the alternative therapy of human squamous cancer A431 in vitro by SPMHT, with high efficacy and no toxicity. Moreover, the optimal conditions found by us (the size and concentration of the magnetic nanoparticles, the amplitude and frequency of the magnetic field, and the duration of treatment) that led to the achievement of high efficiency in the destruction of tumor cells are used in this alternative therapy of squamous cancer.

Thus, for the in vitro therapy of the A431 human epidermoid squamous carcinoma cell line by means of superparamagnetic hyperthermia, we used Fe_3_O_4_-PAA–(HP-γ-CDs) magnetic nanobioconjugates, obtaining very good results, which will be further presented in this paper. The study was performed by using an in vitro model of A431 cell suspension using three concentrations of SPIONs (1, 5, and 10 mg/mL) and employing different magnetic fields of 168, 208, and 370 G, without exceeding the admissible biological limit found experimentally by our group.

## 2. Results and Discussion

### 2.1. Nanobioconjugates Characterization

**Figure 1:**
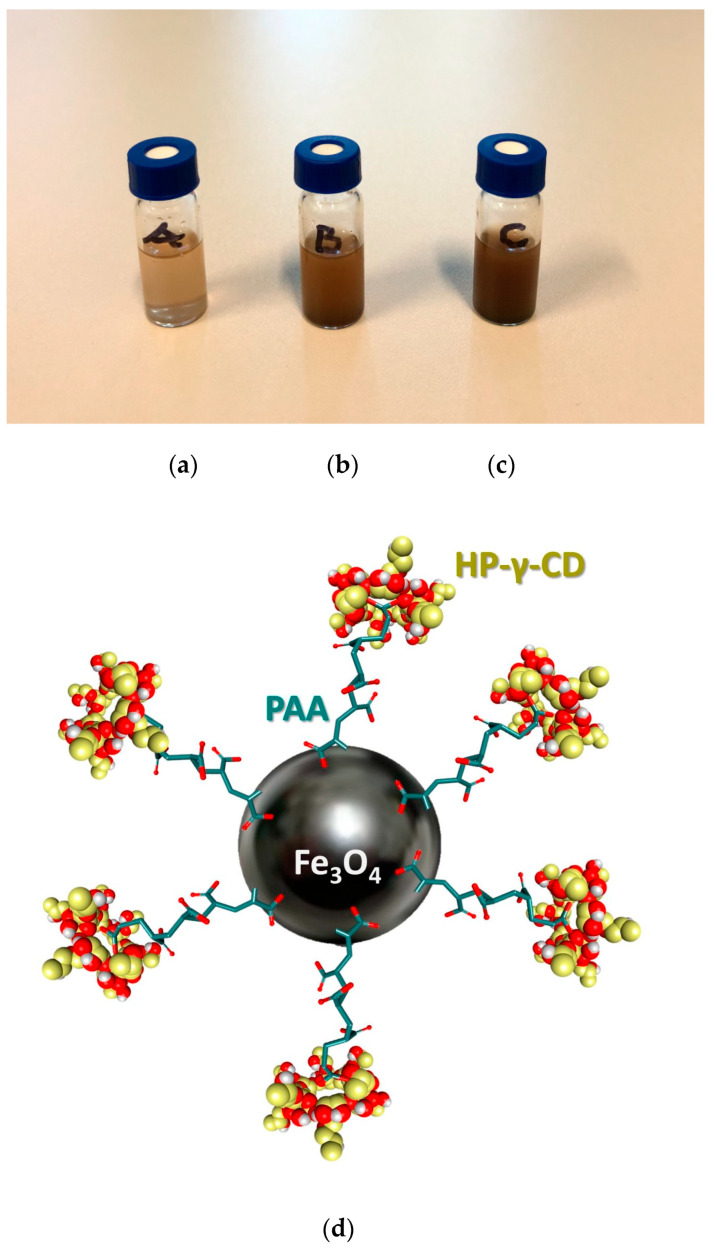
Suspensions of Fe_3_O_4_-PAA–(HP-γ-CDs) nanobioconjugates in PBS (A, B, C) for the concentrations of (**a**) 1, (**b**) 5, and (**c**) 10 mg/mL nanoparticles in a 1 mL of liquid from the vials, and (**d**) the nanobioconjugate structure [22].

The nanobioconjugates used in SPMHT were previously prepared by us and are presented in detail in Ref. [22]. The Fe_3_O_4_-PAA–(HP-γ-CDs) nanobioconjugates (Figure 1) are stable in PBS (phosphate buffer saline with 7.4 pH) for the concentrations of 1 (Figure 1a), 5 (Figure 1b), and 10 mg/mL (Figure 1c) of magnetic nanoparticles in suspension without forming agglomerates, which would lead to their sedimentation (Figure 1a–c).

**Figure 2:**
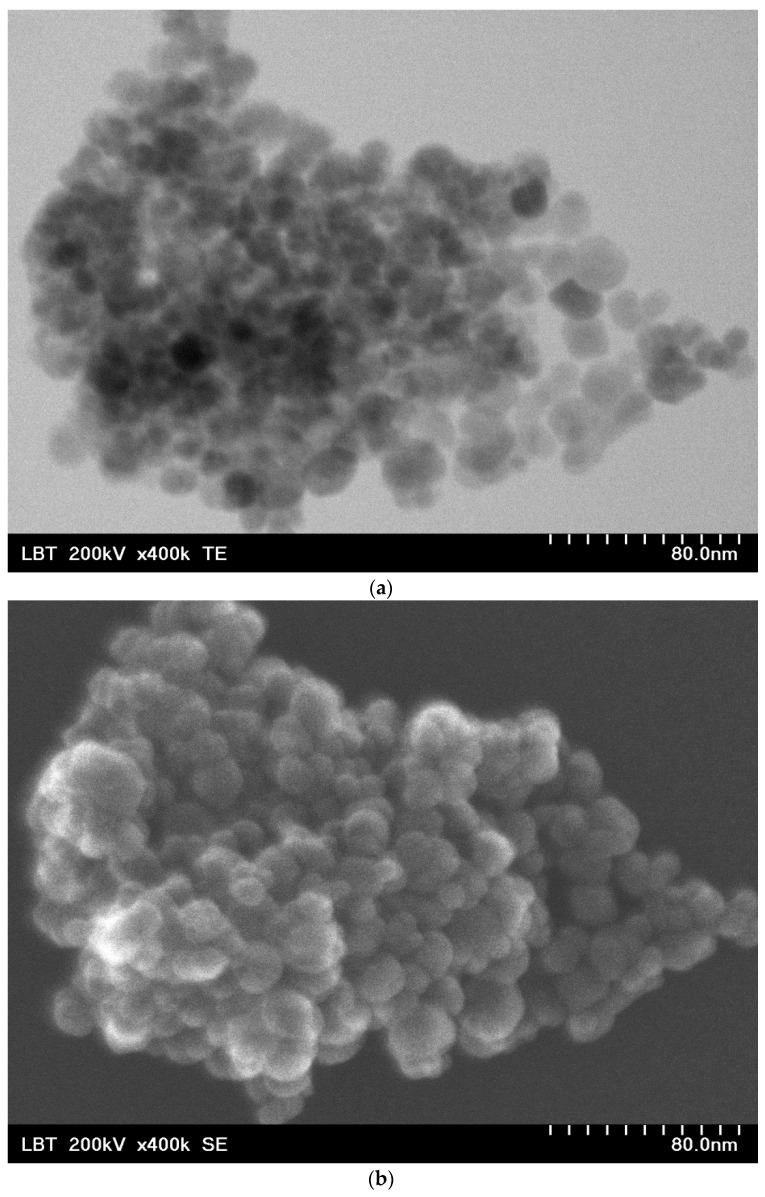

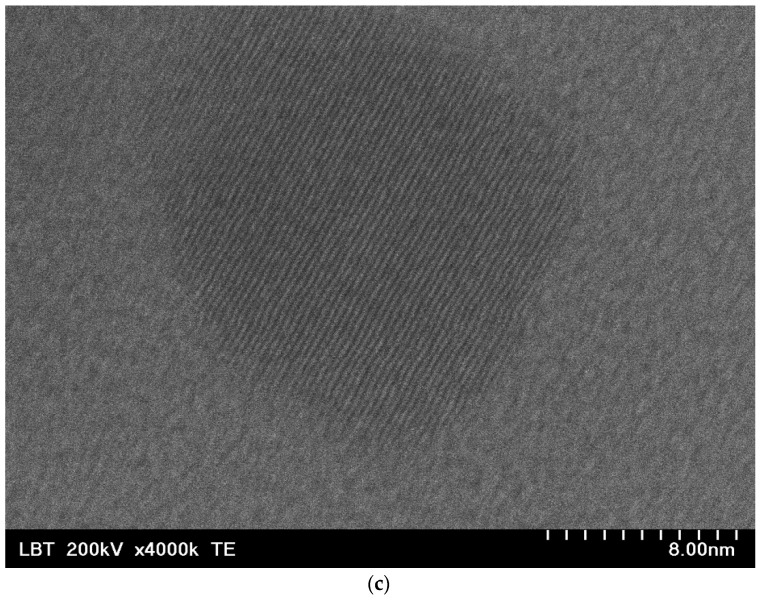
(**a**) TEM and (**b**) SEM images of Fe_3_O_4_ nanoparticles (0.1 nm resolution); (**c**) high-resolution TEM (HR-TEM) image of Fe_3_O_4_ nanoparticle (0.01 nm resolution).

The nanobioconjugates are made of Fe_3_O_4_ (magnetite) nanoparticles with a mean diameter of 15.8 nm, with an approx. spherical shape verified by TEM (Figure 2a) and SEM (Figure 2b) analysis. Nanoparticles are single crystals and monodomain magnetic structures (Figure 2c).

**Figure 3:**
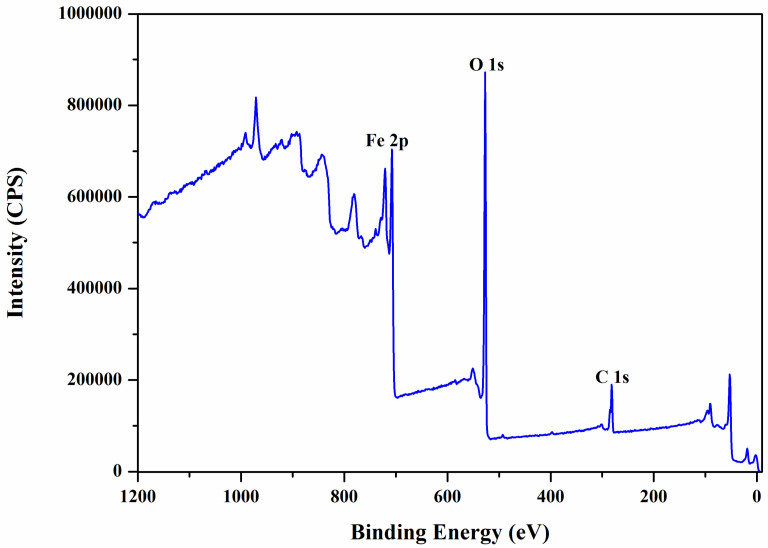
Wide-scan XPS spectra of Fe_3_O_4_-PAA nanoparticles. Acquisition settings: created by Kratos-HP, 300 W X-ray power, XPS monochromatic mode, 20 mA emission current, Al anode library, 1 eV step size scan.

**Figure 4:**
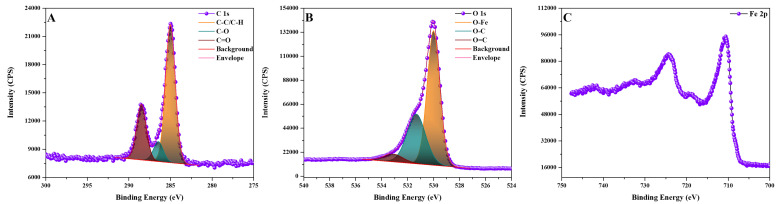
High-resolution XPS spectra of magnetite nanoparticles coated with polyacrylate: (**A**) C 1s; (**B**) O 1s; (**C**) Fe 2p. Acquisition settings: created by Kratos-HP, 300 W X-ray power, XPS monochromatic mode, 20 mA emission current, Al anode library, 0.1 eV step size scan.

Magnetic Fe_3_O_4_ nanoparticles are covered by bioconjugation with hydroxypropyl gamma-cylodextrins (HP-γ-CDs) by means of polyacrylic acid (PAA) biopolymer, also confirmed by XPS (X-ray Photoelectron Spectroscopy) analysis (Figure 3 and Figure 4 and Table 1 and Table 2), in a core-shell nanostructure (core: magnetic nanoparticles, shell: organic layer of PAA–(HP-γ-CDs)).

From the XPS wide-scan spectrum of the sample of magnetite nanoparticles (Fe_3_O_4_) with PAA (C_3_H_4_O_2_), the peaks of iron, carbon, and oxygen (Figure 3 and Table 1) specific to this sample can be observed very clearly. The presence of polyacrylate on the surface of magnetite nanoparticles is confirmed by the appearance of the carbon peak in the spectrum (Figure 3).

High-resolution XPS spectra for the sample (Figure 4 and Table 2) indicate the presence of Fe 2p, C 1s, and O 1s elements in different states.

The peak specific to carbon atoms, C 1s, varies in the 283–292 eV range, and is decomposed into three characteristic bands (Figure 4A) attributed to C-C/C-H, C-O, and C=O bonds; this and confirms the existence of polyacrylate on the surface of magnetite nanoparticles. The peak specific to oxygen atoms, O 1s, varies in the range 528–535.5 eV, and is decomposed into three characteristic bands (Figure 4B), attributed to O-Fe, O-C, and O=C bonds. The O-C and O=C bonds are specific to polyacrylate. Magnetite nanoparticles are proven by the presence of the Fe 2p peak in the 706–736 eV range (Figure 4C).

The XPS analysis revealed both the nature of the magnetite nanoparticles and the presence of polyacrylate on their surface.

The behavior of these nanobioconjugates in an external magnetic field was found to be superparamagnetic (without hysteresis) [28].

The nanobioconjugates with concentrations of 1, 5, and 10 mg/mL were used in in vitro superparamagnetic hyperthermia experiments by suspending them in the cell culture medium. At these concentrations, our previous results showed that healthy cells are not affected [22], and these nanobioconjugates can be safely used in superparamagnetic hyperthermia.

### 2.2. Experimental Conditios in SPMHT with Fe_3_O_4_-PAA–(HP-γ-CDs) Nanobioconjugates: Reaching the Required Temperature for Cancer Cell Therapy

In magnetic hyperthermia therapy, the possible toxicity on healthy cells is mainly due to the magnetic nanoparticles and the magnetic field used. In the case of magnetic nanoparticles, in order to reduce or even eliminate their toxicity on healthy cells, they must be made as biocompatible as possible with the biological environment using different techniques offered by modern nanobiotechnology (by covering them with different biocompatible organic agents, biosurfactation, bioencapsulation in nano-microspheres formed from biological membranes (phospholipids), bioconjugation with different nanobiostructures such as biopolymers or cyclodextrins, etc.) [29,30,31]. However, the biocompatibility is also affected by the concentration of nanoparticles used, even if they are very well-covered or encapsulated in biocompatible nanobiostructures. Data from the literature show that nanoparticle concentrations of up to 20–30 mg/mL can be used, depending on the nature of the magnetic nanoparticles and the biocompatible layer on their surface [32,33].

In the case of our samples, we used gamma-cyclodextrins (more precisely, HP-γ-CDs) to cover ferrimagnetic Fe_3_O_4_ nanoparticles by bioconjugation using the PAA biopolymer [22], which are biocompatible. At the same time, in order to increase the efficiency in magnetic hyperthermia, we proposed the use a concentration of up to 10 mg/mL. This dose was previously tested by us in vitro on healthy human skin cells (HaCaT human keratinocytes) for 30 min, and the cell viability results obtained after 24 h and 48 h showed that they are non-toxic [22]. Thus, in our experiments presented in this paper, we used the safe concentrations of 1, 5, and 10 mg/mL of Fe_3_O_4_-PAA–(HP-γ-CDs) nanobioconjugates to test SPMHT in the therapy of A431 human squamous carcinoma cells (see Section 2.4).

Regarding the magnetic field used in superparamagnetic hyperthermia, it was previously shown that it does not affect healthy cells if the H × f limit (H is the amplitude and f is the frequency of the magnetic field) of 5 × 10^9^ Am^−1^ Hz is not exceeded [21] in the case of surface tumors. However, in magnetic hyperthermia, it is strictly necessary to find the optimal concentration/magnetic field ratio to reach the therapy temperature of ~43 °C and thus obtain a maximum efficiency in hyperthermia. It is always preferable to use lower concentrations of nanoparticles, considering the possible biological implications that they can have on the living organism, in order to ensure a state of safety that guarantees that they do not produce additional cellular toxicity but still allow the therapeutic temperature of 43 °C to be reached in magnetic hyperthermia for an applied magnetic field.

**Figure 5:**
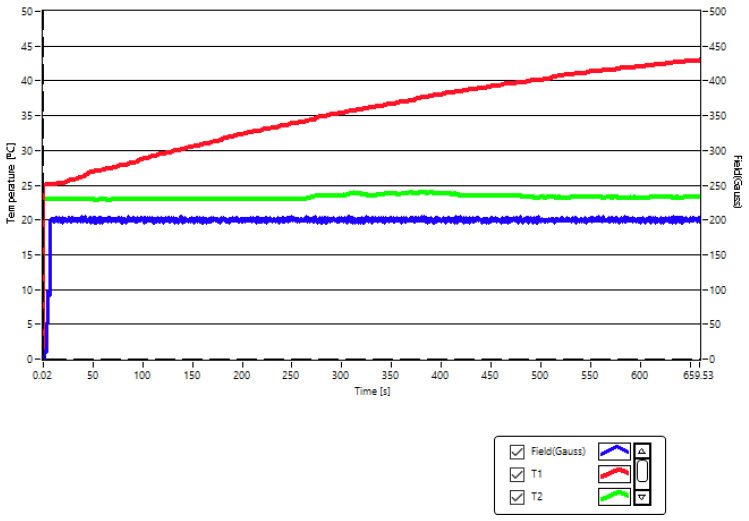
Reaching the therapeutic temperature (T1) in magnetic hyperthermia (43 °C) (red curve) following the application of the magnetic field of 200 G (blue curve) with a frequency of 312.4 kHz on the suspension with the magnetic nanobioconjugates of Fe_3_O_4_-PAA–(HP-γ-CDs) having a concentration of 5 mg/mL in PBS. The experiment was carried out in adiabatic conditions. The recorded green curve shows the room temperature (T2). Observables accuracy: 1 G for magnetic field, 0.1 °C for temperatures, 0.01 kHz for magnetic field frequency.

Thus, in our case, for a concentration of 5 mg/mL of Fe_3_O_4_-PAA–(HP-γ-CDs) nanobioconjugates dispersed in PBS suspension (5 mg nanobioconjugates in 1 mL of PBS), in order to reach the temperature of 43 °C necessary for the therapy, we had to use a magnetic field of 200 G (15.92 kA/m in SI units) at a frequency of 312.4 kHz (Figure 5). In this case, the therapy temperature (red curve (T1)) is reached in a short time of approx. 11 min.

However, this field is very close to the maximum biological limit allowed (5 × 10^9^ Am^−1^ Hz), its parameters leading to the value H × f = 4.992 Am^−1^ Hz. Moreover, for the lower concentration of nanoparticles of 1 mg/mL, the magnetic field had to be increased to the value of 370 G (29.44 kA/m) at the same frequency of 312.4 kHz in order to be able to reach the therapy temperature of 43 °C in magnetic hyperthermia. However, in this case, the known admissible biological limit would be exceeded, with the magnetic field parameters reaching the value of ~9 × 10^9^ Am^−1^ Hz. Considering this result and the previously known limitation [21], it was necessary in our case to first check if the new values of the magnetic field amplitude of 200 and 360 G for the frequency of 312.4 kHz, which were to be used in our superparamagnetic hyperthermia experiment for the in vitro therapy of human squamous skin carcinoma (A431), corresponding to the two concentrations of 1 and 5 mg/mL, would affect the cells of A431 or not. These aspects are presented in the next section.

### 2.3. Squamous Skin Carcinoma (A431) Cells Viability above the Known Biological Magnetic Field Limit

**Figure 6:**
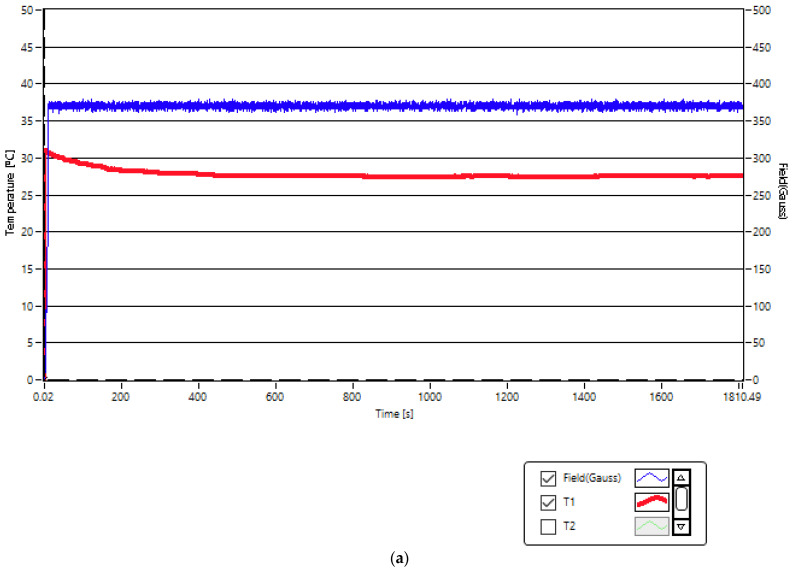

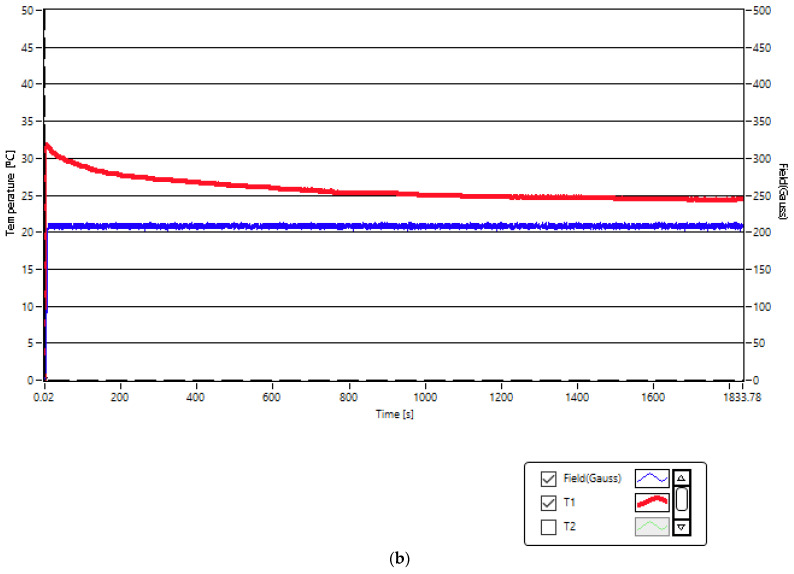
The experimental curves recorded for the magnetic fields (blue curves) and the temperatures in the A431 cell culture (red curves (T1)) for a duration of 30 min of the application of magnetic fields of (**a**) 370 G and (**b**) 208 G. Accuracy: 1 G for magnetic field, 0.1 °C for temperature.

First, before the application of superparamagnetic hyperthermia therapy on A431 cancer cells using nanobioconjugates of Fe_3_O_4_-PAA–(HP-γ-CDs) in the established concentrations of 1, 5 and 10 mg/mL, we tested the possible toxicity of the magnetic field on the A431 cells for the values that exceed the allowed limit, respectively, for the field of 208 and 370 G corresponding to concentrations of 1 and 5 mg/mL. It should be mentioned that the field values had to be higher than the known biological limit in order to be able to obtain the optimal temperature for therapy of 43 °C in the case of A431 cancer cells in suspension. For this, we used in the experiment the values of the parameters from Table 3 for the concentrations that require the use of higher magnetic fields. The experiment was carried out at room temperature (~25.5 °C) by applying the magnetic fields for approx. 30 min, as shown by the results recorded in Figure 6a,b (blue curves). This duration will be used later (Section 2.4) in the superparamagnetic hyperthermia of cancer.

The experimental results shows that the temperature recorded in the A431 cell culture (red curves) when magnetic fields are applied does not increase but stabilizes around the room temperature. The experiment also confirms the fact that magnetic hyperthermia (increasing the temperature in the cellular environment to 43 °C) cannot be obtained in the absence of magnetic nanoparticles by only applying the magnetic field. However, in the case of the higher magnetic field (370 G) (Figure 6a), a small increase in temperature (~2 °C) is observed in the cell culture that is most probably due to the eddy currents that appear at 312.4 kHz frequency in the conductive cell medium as a result of the significantly higher value of this field compared to the 200 G field (Figure 6b).

**Figure 7:**
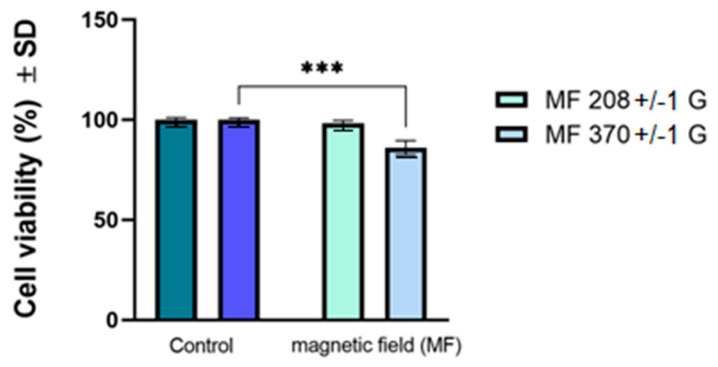
Cell viability of human squamous cell carcinoma (A431) after exposure to magnetic field (MF) for 30 min at frequency (f) of 312.4(±0.01) kHz and two amplitudes (H) of 208(±1) and 370(±1) G. Cell viability was determined at 24 h post-exposure to magnetic field and normalized to control cells (cells cultured with specific growth medium under standard conditions (37 °C)). Results are presented as mean values ± standard deviation (SD). One-way ANOVA analysis was performed to evaluate the statistical differences followed by Tukey’s multiple comparisons tests (*** *p* < 0.001).

Figure 7 and Table 4 present the results of cell viability obtained for the A431 human carcinoma cell line after exposure to a magnetic field (frequency of 312.4 kHz and amplitudes of 208 and 370 G). The data show that the magnetic field parameters induced a moderate decrease in the viability of A431 cells compared to the cells maintained under standard conditions at 37 °C, obtaining viabilities of 98.3% and 86%, depending on the magnetic field amplitude. However, the cell viability rates exerted by the A431 cells are within the limit accepted by ISO standards (until minimum 70%) (International Standard Organization, Geneva) [34]; therefore, these parameters of the magnetic field were further used to obtain magnetic hyperthermia when the cells were exposed to different concentrations of Fe_3_O_4_-PAA–(HP-γ-CDs) nanobioconjugates.

The cell viability results presented in Figure 7 and Table 4 are also endorsed by the cell density assessment, as depicted in optical microscopy images from Figure S1 (Supplementary Material). The images were captured using 10× magnification by using the bright field illumination setup and employing an inverted microscope from Optika Microscope Optikam Pro Cool 5 and Optika View software (Ponteranica, BG, Italy).

Figure S1 shows the cell density of A431 cells under standard conditions (37 °C) and post-exposure to different amplitudes of the magnetic field (208 and 370 G). Thus, it can be observed that by comparison with standard cells (A), both types of cells maintained under laboratory conditions (B) and the cells exposed to the magnetic field (C) did not show a decrease in cell density; however, the morphology of the cells was slightly modified 24 h post-exposure to the test parameters. For the concentration of magnetic nanoparticles of 10 mg/mL from Fe_3_O_4_-PAA–(HP-γ-CDs) nanobioconjugates dispersed in the culture medium and subjected to the smaller magnetic field of 168 G (13.37 kA/m), which is sufficient in this case to reach the temperature of 43 °C therapy, no cell density changes were found in the A431 cells, with cell viability being close to 100%.

Thus, the concentrations of nanoparticles of 1, 5, and 10 mg/mL and the corresponding magnetic fields of 370, 208, and 168 G to reach the therapy temperature of 43 °C can be safely used for the in vitro therapy of human squamous skin carcinoma by superparamagnetic hyperthermia using nanobioconjugates of Fe_3_O_4_-PAA–(HP-γ-CDs).

### 2.4. In Vitro Squamous Skin Carcinoma Cells Therapy by Superparamagnetic Hyperthermia with Fe_3_O_4_-PAA–(HP-γ-CDs) Nanobioconjugates

**Figure 8:**
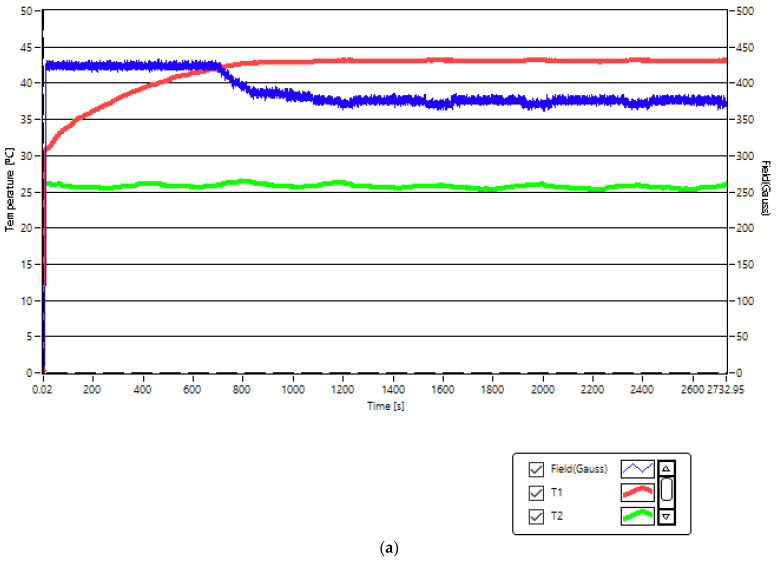

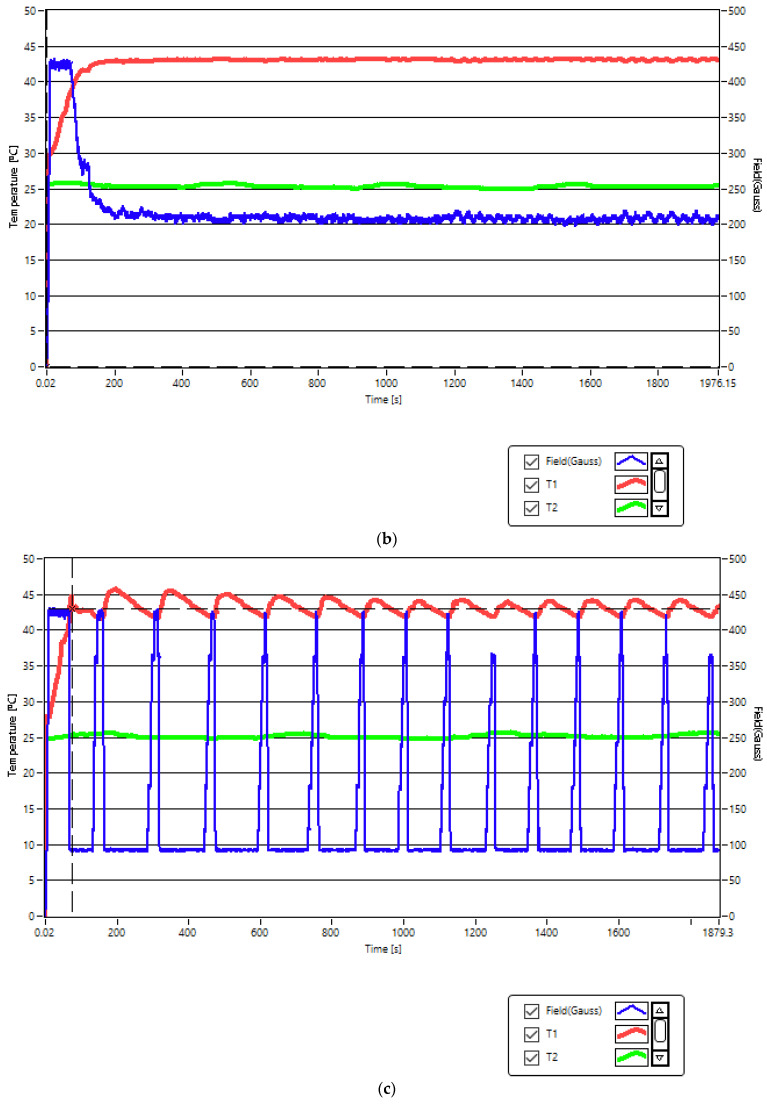
Temperatures (T1—red curves) and duration of therapy for the concentrations of magnetic nanoparticles from Fe_3_O_4_-PAA–(HP-γ-CDs) nanobioconjugates and magnetic fields of (**a**) 1 mg/mL and 370 G, (**b**) 5 mg/mL and 208 G, and (**c**) 10 mg/mL and 168 G. Observables accuracy: 1 G for magnetic field, 0.1 °C for temperatures.

The in vitro therapy of A431 human epidermoid squamous carcinoma through superparamagnetic hyperthermia with nanobioconjugates of Fe_3_O_4_-PAA–(HP-γ-CDs) having magnetic nanoparticle concentrations of 1, 5, and 10 mg/mL in the culture medium was performed for 30 min at an average temperature of ~43 °C (Figure 8) by using magnetic fields of 370 G for the concentration of 1 mg/mL (Figure 8a), 208 G for the concentration of 5 g/mL (Figure 8b), and an equivalent pulsating magnetic field on average of a continuous field of approx. 168 G for the concentration of 10 mg/mL (Figure 8c). The therapy temperature (T1) reached in the experiment is shown in Figure 8 by the red curves, compared to the room temperature, shown by the green color (T2), which remains approx. constant (25–26 °C). The duration until reaching the therapy temperature of 43 °C after the application of the magnetic field depends on the concentration of the magnetic nanoparticles in the samples used, 1.08 min being the lowest in the case of the sample with a concentration of 10 mg/mL, respectively; of 2.5 min in the case of the 5 mg/mL sample; and 14 min in the case of the 1 mg/mL sample. Thus, in all cases, the therapy temperature in SPMHT is reached quite quickly, even in the case of the most diluted sample (1 mg/mL), with these samples being very suitable for use in this type of experiment.

The pulsating field (harmonic alternative and applied periodically for short periods of time) was used in the experiment to see if it would bring additional benefits from the point of view of the effect of magnetic hyperthermia on tumor cells. The result is presented in Section 2.5. Moreover, the fact that the field should not exceed the maximum admissible biological limit compared to the case of its continuous application was taken into account in this case.

After the completion of the in vitro therapy experiment (30 min after reaching the temperature of 43 °C), the cell cultures were incubated for 24 h in the standard culture medium, and then they were analyzed by the Alamar blue test (Section 3.4). The results obtained are presented in the next Section 2.5.

### 2.5. Biological Impact of Fe_3_O_4_-PAA–(HP-γ-CDs) Nanobioconjugates under SPMHT Conditions on A431 Cell Viability

**Figure 9:**
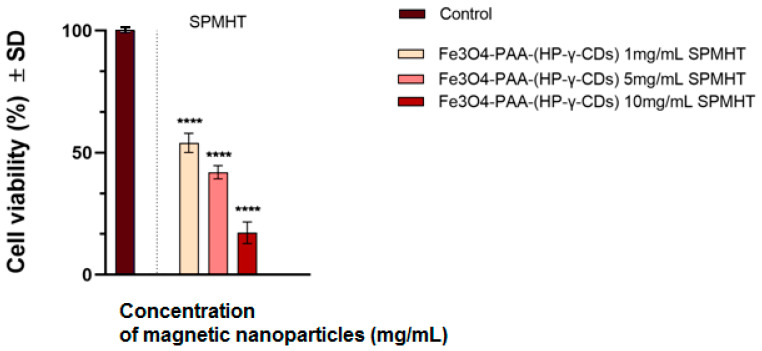
Evaluation of cell viability induced by magnetic nanoparticles in suspension under superparamagnetic hyperthermia (SPMHT) for the human squamous carcinoma cell line (A431). Cell viability was normalized to control cells (cells cultured with specific growth medium under standard conditions (37 °C)). Results are presented as mean values ± standard deviation (SD). One-way ANOVA analysis was performed to evaluate the statistical differences followed by Dunnett’s multiple comparisons tests (**** *p* < 0.0001).

Figure 9 shows the viability of treating the A431 human carcinoma cell line with Fe_3_O_4_-PAA–(HP-γ-CDs) magnetic suspension at different concentrations (1, 5, and 10 mg/mL) when superparamagnetic hyperthermia (SPMHT) is employed. In the case of the concentration of 10 mg/mL, the pulsating magnetic field was applied (Figure 8c), compared with the concentrations of 1 and 5 mg/mL, where the magnetic field was applied continuously (Figure 8a,b) for 30 min. The results reveal that the human skin carcinoma (A431) cells treated with Fe_3_O_4_-PAA–(HP-γ-CDs) at concentrations of 1, 5, and 10 mg/mL and subjected to SPMHT (43 °C for 30 min) presented decreased viability rates corresponding to 54.04, 42.01, and 17.2%. Thus, it can be stated that the induction of SPMHT significantly contributes to the reduction of cell viability; the highest difference of 82.8% in cell viabilities (the percentage of cancer cells destroyed) relative to the viability of cells cultured under standard conditions without application of SPMHT was observed for the highest concentration of magnetic nanoparticles (10 mg/mL).

Cell viability percentages with their standard deviations (SDs) of A431 cells treated with Fe_3_O_4_-PAA–(HP-γ-CDs) nanobioconjugates under SPMHT are presented in Table 5.

In addition, in the case of the sample with a concentration of 10 mg/mL where a pulsating magnetic field was used, it can be seen that it would bring additional benefits for magnetic hyperthermia because cell viability decreased substantially in this case (by ~25 percent); however, the concentration of nanoparticles increased only twice and the magnetic field decreased by only 32 G compared to the sample with a concentration of 5 mg/mL (Figure 9), whereas the cell viability induced by this decreased significantly less (by only ~12 percent) than in the case of the 1 mg/mL sample, its concentration being five times higher. However, this aspect will be much better elucidated through our future studies where we will separately consider the effect of the concentration of nanoparticles, as well as the continuous or pulsating application of the magnetic field on the viability of tumor cells following superparamagnetic hyperthermia, in order to identify much more clearly the effects induced by these parameters (concentration, continuously applied harmonic alternating magnetic field, and pulsating applied harmonic alternating magnetic field (in short time intervals)) on tumor cells.

Previously, we obtained similar results in the case of MCF-7 breast cancer cell therapy [28], where the destruction of tumor cells was at a significantly higher percentage of 95.11% for the nanoparticles concentration of 10 mg/mL under the same temperature conditions (43 °C) and duration of treatment (30 min). This result shows that the efficacy of superparamagnetic hyperthermia therapy with Fe_3_O_4_-PAA–(HP-γ-CDs) nanobioconjugates depends on the type of tumor cells. Thus, the MCF-7 breast cancer cells are more sensitive to the hyperthermic effect than A431 human epidermoid squamous carcinoma cells.

Other authors have recently obtained similar results in the case of squamous carcinoma or other skin cancers (both in vitro and in vivo) using magnetic hyperthermia with biocompatible magnetite (Fe_3_O_4_) nanoparticles (Table 6) [35,36,37,38].

Thus, Legge et al. [35], using 8 nm magnetite nanoparticles coated with a 6 nm amine-terminated silica shell and functionalized with αvβ6 antibodies in a magnetic field of 9.7 mT for 10 min, obtained a reduction of below 50% cell viability of the VB6 cell lines compared to that of the control at 24 h post-treatment and below 25% after 48 h.

Su et al. [36] obtained in vivo a tumor inhibition of ~33% in male BALB/c nude mice after applying magnetic hyperthermia using anti-CD44 antibody-modified Fe_3_O_4_ nanoparticles for 30 min at the frequency of 237 kHz and the current of 50 A in the induction coil.

The following case is an example of using magnetic hyperthermia in skin cancer therapy with magnetic nanoparticles of Fe_3_O_4_. Aiming for the elimination of toxicity in this type of therapy, as well as a more homogeneous distribution of magnetic nanoparticles at the level of the tumor for its uniform heating, Ref. [37] shows another variant of magnetic hyperthermia: by applying bandages on the tumor skin, made of polycaprolactone (PCL) fibers containing magnetic Fe_3_O_4_ nanoparticles with a size of 50–100 nm. Through this procedure, the magnetic nanoparticles are no longer introduced into the tumor, being located only on the surface of tumor where the bandage is found, thus avoiding their possible toxicity on the healthy tissue. The results obtained in this way, both in vitro and in vivo, on skin cancer show the viability of this technique which, in addition to its high effectiveness on the tumor, greatly reduces the duration of therapy and post-treatment. Thus, the viability of cancer cells can be reduced to ~50% after 2 h post-treatment, and in vivo on BALB/c mice, the tumor can be reduced by ~82% after two consecutive treatment sessions (at 2 days).

Previously, Baldi et al. [38] used Fe_3_O_4_-1-PNPs-hEGFR magnetic nanoparticles (PNPs: polymeric nanoparticles, hEGFR: human epidermal growth factor receptor) with a size of ~12 nm (for Fe_3_O_4_) and a concentration of 4.5 mg/mL for treatment by magnetic hyperthermia in vivo and obtained a 60% decrease in volume of A431 tumors in SCID mice.

In order to be able to compare easily, these results, the results obtained by us, and the conditions used in magnetic hyperthermia are quantitatively and synthetically shown in Table 6.

However, in comparison, the result obtained by us in vitro for the sample with the concentration of 10 mg/mL is seen to be significantly better (17.2% viability of A431 cells) due to our optimal conditions used in the therapy (magnetic field, frequency, size of magnetic nanoparticles, concentration of nanoparticles, bioconjugation with HP-γ-CDs, and duration of therapy) and the type of cancer cells used in the superparamagnetic hyperthermia experiment.

As can be seen from Table 6, the cancer results obtained in the magnetic hyperthermia depend a lot on these parameters. Thus, the high efficacy of this therapy and the lowest possible toxicity (or even no toxicity) is a target to pursue in this type of alternative cancer therapy, both in vitro and in vivo, in order to be applied in clinical trials in the future.

### 2.6. SPMHT Limitations

Magnetic hyperthermia in cancer therapy is a very promising alternative therapy but, at the same time, is still limited in application related to: the type of magnetic hyperthermia (MHT/SPMHT) [2,8,9], the alternating magnetic field that can be used in therapy [21], the magnetic nanoparticles [7,8,14], the biocompatibility of magnetic nanoparticles with the biological environment versus their possible toxicity [10], and even the conditions for the practical implementation of magnetic hyperthermia in cancer therapy, both in vitro and in vivo, even in clinical trials [1,2,32,39,40,41]. Thus, MHT is seen to be less efficient than SPMHT in terms of the SLP (specific loss power) that leads to the heating of the magnetic nanoparticles, which is significantly lower. However, even in the case of SPMHT, which is more efficient [14], this loss power depends very much on the size of the magnetic nanoparticles used, which is a critical parameter [9]. Therefore, for SPMHT to be effective, an optimal ratio must be found between obtaining the maximum SLP and the size of the magnetic nanoparticles so that the nanoparticles do not produce cellular toxicity. Moreover, the SPMHT efficiency depends a lot on the harmonic alternating magnetic field used, the amplitude (H), and frequency (f) of the field, respectively. SLP increases significantly with H^2^ but also with f. However, the magnetic field used in SPMHT is limited in amplitude and frequency so as not to affect healthy cells [21]. Thus, in SPMHT, the amplitude of the magnetic field must not exceed 30–40 kA/m, both to remain in the field of superparamagnetic relaxation in magnetic nanoparticles and to not affect healthy tissues. In addition, the frequency cannot be high, although this would be advantageous from the point of view of SLP and, implicitly, of the quick heating of the magnetic nanoparticles to reach the temperature required for therapy (~43 °C). However, the maximum permissible biological limit in vivo would be H × f = 5 × 10^9^ Am^−1^ Hz [21], or the one we found in vitro in this work for SPMHT, which is 9.2 × 10^9^ Am^−1^ Hz (for the field maximum of 370 G and frequency of 312.4 kHz in the case of the sample with a concentration of 1 mg/mL), without cellular damage. Regarding magnetic nanoparticles used in SPMHT, magnetite (Fe_3_O_4_) was found to be much more suitable than other nanoparticles we also used in this work, both from the point of view of cytotoxicity (this being much reduced), as well as its magnetic properties, which are very suitable for obtaining a powerful magnetic hyperthermia (high-saturation magnetization, good initial magnetic susceptibility, and low magnetic anisotropy) [9]. Moreover, in SPMHT, in addition to the nature of the nanoparticles and their size, both their shape and size distribution are important, as they can limit the hyperthermic effect. A wide size distribution of magnetic nanoparticles significantly decreases SLP [9,42] (or SAR (specific absorption rate)). In addition, a shape of the magnetic nanoparticles different from the spherical one (e.g., elongated or ellipsoidal) increases their magnetic anisotropy, which has a direct effect on the decrease of the critical size (diameter) for the nanoparticles and, implicitly, of the SLP [9]. In our experiment, we used rather spherical nanoparticles with a not-too-wide distribution, thus finding the optimal size (average diameter) of Fe_3_O_4_ nanoparticles to be 15.8 nm (Figure 2) to obtain the maximum SLP [22]. However, in order to not produce toxicity on healthy cells, magnetic nanoparticles must be made biocompatible with the biological environment, especially when they are large (>10 nm) and are used in high concentrations (mg/mL). This will greatly influence the results of the SPMHT therapy so that it can be obtained with as low toxicity as possible or even without toxicity, as we previously obtained by using PAA-(HP-γ-CDs) to cover Fe_3_O_4_ magnetic nanoparticles [22]. A cellular toxicity or a viability of nanoparticles (covered with any organic layer), respectively, below 70% is unacceptable in SPMHT, according to ISO standards [34]. So, another limitation in SPMHT is given by the possible cytotoxicity obtained in the case of magnetic nanoparticles covered with different organic agents through biosurfactation, bioencapsulation, bioconjugation, or biofunctionalization. Therefore, prior testing of nanobioparticles in the biological environment is necessary before using them in SPMHT for cancer therapy. In addition to all the above aspects, the conditions used for the implementation of SPMHT therapy with biocompatible magnetic nanoparticles are also important. Thus, in SPMHT, there are also limitations regarding the dose of nanoparticles and the duration of the therapy used in the treatment of the tumors. If they are not found suitable for therapy, they affect healthy cells. In our experiments, we found the appropriate dose to be up to 10 mg/mL, which leads to maximum effectiveness and for which the viability of healthy HaCaT human keratinocyte cells is around 100% [22]. Moreover, the therapy duration of 30 min found by us is reasonable. A long duration of over an hour can affect healthy cells [43,44,45].

Meanwhile, several studies have already sustained that magnetically induced hyperthermia has been successfully employed in several pre-clinical studies. Gupta and Sharma [46] revealed not long ago that this method was able to increase BBB permeability, hence increasing drug concentrations for glioblastoma therapy. Another study correlates the use of hyperthermia and classical chemotherapeutics in combating drug resistance, providing a synergistic therapeutic effect to this type of treatment strategy [47,48]. Therefore, no alternative treatment strategy should be downplayed, especially when referring to cancer treatment approaches. Therefore, within its limitations, hyperthermia should be considering a promising alternative strategy which should upscale the development of the upcoming anti-cancer-orientated therapies.

## 3. Materials and Methods

### 3.1. The Human Epidermoid Squamous Carcinoma Cell Line

The human epidermoid squamous carcinoma cell line (A431) was acquired from the American Type Culture Collection (ATCC, Manassas, VA, USA) as a frozen item. The A431 squamous cancer cell line was cultured in Dulbecco’s Modified Eagle’s Medium modified to contain 4 mM L-glutamine, 4500 mg/L glucose, 1 mM sodium pyruvate, and 1500 mg/L sodium bicarbonate which was further supplemented with 10% fetal bovine serum (FBS). An antibiotic mixture of penicillin/streptomycin (100 U/mL penicillin and 100 U/mL streptomycin) was added into the medium to prevent microbial contamination. The cell cultures were maintained under standard conditions, employing a humidified atmosphere with a relative humidity (RH) value of 95% (to minimize media evaporation and condensation) and 5% CO_2_; also, a temperature of 37 °C was assured by using a Steri-Cycle i160 incubator (Thermo Fisher Scientific, Inc., Waltham, MA, USA).

### 3.2. Nanobioconjugates Used in SPMHT for Cancer Therapy

In the in vitro therapy of A431 squamous cancer using superparamagnetic hyperthermia, the SPIONs-PAA–(HP-γ-CDs) nanobioconjugates previously obtained by us [22] were used, where SPIONs are Fe_3_O_4_ (magnetite) nanoparticles. First, the Fe_3_O_4_ magnetic nanoparticles were synthesized by the chemical co-precipitation method. Then, the magnetic nanoparticles were covered with polyacrylic acid (PAA) (step two) to be finally conjugated with hydroxy-propyl-γ-cyclodextrins (HP-γ-CDs) (step three), thus obtaining the Fe_3_O_4_-PAA–(HP-γ-CDs) nanobioconjugates. The nanobioconjugates were obtained by using synthesized Fe_3_O_4_-PAA nanoparticles and commercially available 2-hydroxy-propyl-γ-cyclodextrins (HP-γ-CDs) in a molar ratio of 1:1. The complete synthesis method is presented in Ref. [22]. From the masses of nanobioconjugates, taking into account the molar masses of the chemical components, the mass of Fe_3_O_4_ magnetic nanoparticles was determined to obtain dispersions in phosphate buffer saline (PBS) of 1, 5, and 10 mg/mL (the concentrations of Fe_3_O_4_ nanoparticles from the nanobioconjugates (Fe_3_O_4_-PAA–(HP-γ-CDs) in a 1 mL PBS). These concentrations of magnetic nanoparticles from nanobioconjugates are used in SPMHT experiments.

### 3.3. In Vitro SPMHT Experiment

In vitro superparamagnetic hyperthermia of A431 squamous cancer cells was performed using the F3 Driver magnetic hyperthermia generator having the power of 3 kW, cooled with deionized water in a closed circuit. This driver is provided with a special inductor coil for carrying out experiments in adiabatic conditions (without heat loss to the outside) [22]. The monitoring of the experiment was performed by means of its own Maniac (‘21) professional software and PC using a data acquisition system.

The precision in measuring the magnetic field was 1 G. The temperature during the magnetic hyperthermia experiment was measured with an accuracy of 0.1 °C using a precision fiber optic thermometer.

The generator allows for keeping the temperature constant at a value of 42.9–43 °C during the experiments through the automatic tune setting (by electronic feedback loop) of the magnetic field amplitude.

The duration of the superparamagnetic hyperthermia experiments for the therapy was 30 min.

### 3.4. Alamar Blue Colorimetric Test

To assess the cytotoxicity of Fe_3_O_4_-PAA–(HP-γ-CDs) suspension under magnetically induced hyperthermia, a protocol developed by our team and published in detail in another recent article [28] was performed.

Alamar blue assay was used to assess the cytotoxicity of Fe_3_O_4_-PAA–(HP-γ-CDs) suspension under magnetically induced hyperthermia described in detail elsewhere [28] by employing cell suspensions of 1 × 10^5^ cells/mL. The cytotoxicity of Fe_3_O_4_-PAA–(HP-γ-CDs) sample at three different concentrations (1, 5, and 10 mg/mL) on A431 human epidermoid squamous carcinoma cell suspension was evaluated.

In brief, post-exposure to AMF, the cell suspension was seeded in 96-well plates using a density of 2 × 10^4^ cells/well. The plates were further incubated in a humidified atmosphere at 37 °C and 5% CO_2_ for 24 h, and afterwards, the cells were washed with PBS (three times) to avoid a possible interference with Alamar blue reagent, as previously reported [22]. Control cells were considered the cells treated with culture media, under standard conditions (37 °C). A stock solution of 0.01% Alamar blue in PBS (*w*/*v*) was further added on each well for cell viability quantification. The mechanism underlying this method consists in the conversion of the initially weak fluorescent blue dye resazurin salt to a highly fluorescent pink compound (resorufin) by the metabolically active cells. Thus, by changing the color, the percentage of viable cells could be spectrophotometrically quantified by using the following formula:

$$Cell\;viability\;(\%)=\frac{{(\varepsilon }_{OX})\lambda_{2}\;\cdot A_{test}\;\lambda_{1}-\left(\varepsilon_{OX}\right)\lambda_{1}\cdot A_{test}\;\lambda_{2}}{\left(\varepsilon_{OX}\right)\lambda_{2}\cdot A_{0}\lambda_{1}-\left(\varepsilon_{OX}\right)\lambda_{1}\cdot A_{0}\lambda_{2}}\times 100$$

where *ε_ox_* is the molar extinction coefficient of the oxidized Alamar blue reagent, *A_test_* is the absorbance of test wells, *A*_0_ is the absorbance of the control well, *λ*_1_ = 570 nm, and *λ*_2_ = 600 nm.

The absorbances were determined at two different wavelengths (570 nm and 600 nm) at 3 h post-addition of the Alamar blue reagent by using a microplate reader (xMark^TM^ Microplate, Bio-Rad Laboratories, Hercules, CA, USA), as previously described [28].

**Figure 10:**
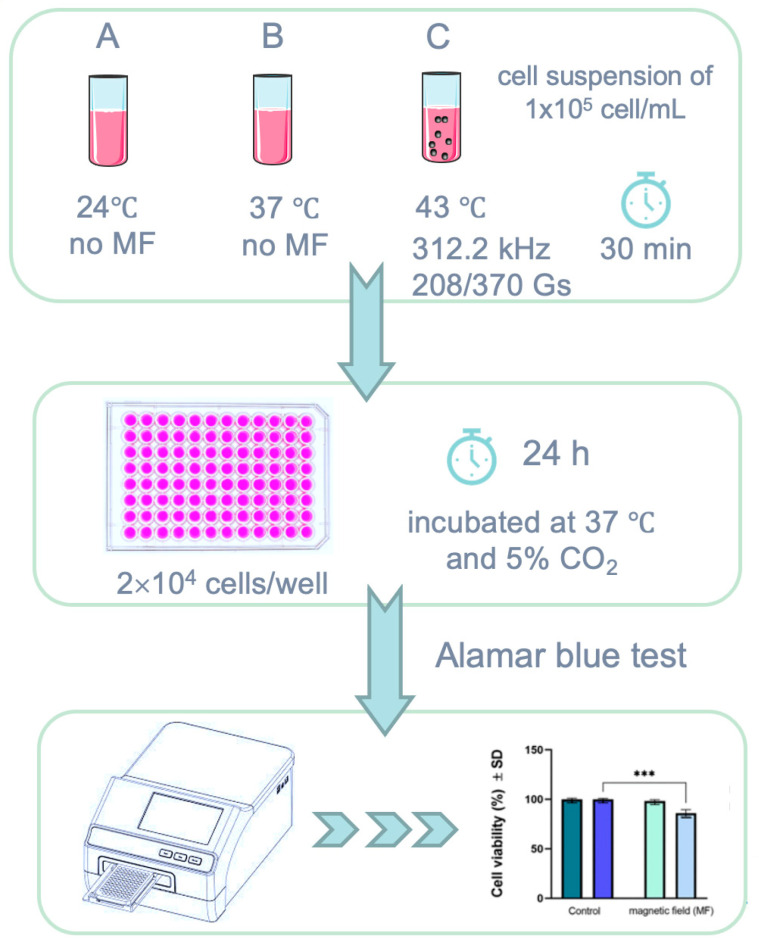
Schematic representation of cell viability assessment via Alamar blue method employing magnetically induced hyperthermia: (**A**) under standard conditions (37 °C), (**B**) under laboratory conditions (24 °C), and (**C**) after exposure to magnetic field (MF) for 30 min at frequency of 312.4(±0.01) kHz and amplitudes of 208(±1) and 370(±1) G; *** *p* < 0.001.

A schematic representation of the methodology employed for the in vitro assessments is provided below (Figure 10).

### 3.5. Representation and Statistical Evaluation of Experimental Data

GraphPad Prism 9 version 9.3 (GraphPad Software, San Diego, CA, USA) was used for data representation and statistical analysis. The results are presented as mean values ± standard deviation (SD). One-way ANOVA was performed to obtain the statistical differences, followed by Tukey’s or Dunnett’s multiple comparison post-test, as was done previously [28].

## 4. Conclusions

In this paper, we showed that superparamagnetic hyperthermia with nanobioconjugates of Fe_3_O_4_-PAA–(HP-γ-CDs) was successfully applied in vitro for the therapy of human squamous cancer A431 under the optimal conditions established by us: magnetic nanoparticle concentrations of 1–10 mg/mL, a field amplitude of 168–370 G at a frequency of 312.4 kHz, and a treatment duration of 30 min; to increase the efficacy and cellular non-toxicity. Thus, the A431 tumor cells are destroyed in a high percentage of 82.8% after 24 h from the application of the treatment in the case of the nanoparticle concentration of 10 mg/mL and the magnetic field of 168 G. In this case, the parameters of the magnetic field (frequency of 312.4 kHz and amplitude of 168 G (13.37 kA/m)) are in the allowed range for biological safety (H × f < 5 × 10^9^ Am^−1^ Hz): H × f = 4.18 × 10^9^ Am^−1^ Hz.

In addition, the percentage of tumor cell destruction could be increased by repeating the therapy after a time interval of 24–48 h for the destruction of resistant tumor cells remaining after the first treatment, as shown in Ref. [35] for in vitro magnetic hyperthermia.

Compared to other recent results obtained by other authors for in vitro skin cancer therapy through magnetic hyperthermia with Fe_3_O_4_ nanoparticles (Table 4), we think that the very good result obtained by us of tumor cell viability at the percentage of only 17.2% is due to the optimal conditions previously found by us and applied in this therapy: Fe_3_O_4_ nanoparticle size, bioconjugation of nanoparticles with biocompatible HP-γ-CDs, concentration of nanoparticles in suspension (10 mg/mL), parameters of the magnetic field (H,f), temperature, and duration of therapy. Thus, the results obtained by us are very promising for the future use of this therapy in vivo on the animal model in the effective and non-toxic treatment of squamous cancer.

In conclusion, for an effective therapy of tumors, the SPMHT limits must be taken into account when finding all the above conditions which must be optimal (without exceeding the limits) for the effective application of the therapy, both in vitro (as is the case of this work) as well as in vivo, in order to obtain the maximum efficacy (over 90%) in the destruction of tumor cells and minimal toxicity on healthy cells, or even no toxicity.

Based on the results obtained so far in this field [1,2,8,28,32,41], it can be concluded that the method of alternative therapy through SPMHT using biocompatible magnetic nanoparticles of Fe_3_O_4_-PAA–(HP-γ-CDs) SPIONs nanobioconjugates can be very effective in the early destruction of localized tumors (before they form metastases in the body), keeping in mind that the cancer hijacks the body’s homeostasis by the neuroendocrine and immune systems, setting the body in a homeostatic mode favoring the tumor’s expansion that affects the body and brain functions [49]. However, in the future, after the advancement of research in this field, it could be possible to use SPMHT in the therapy of tumors extended in the body (metastases) by using biocompatible and biofunctionalized SPIONs magnetic nanoparticles (using modern nanobiotechnology) with agents specific to tumor cells (type markers, monoclonal antibodies, etc.) that target tumor cells in the body. Then, by applying the magnetic field to the whole body, these spreading tumor cells can be destroyed.

## Figures and Tables

**Table 1:** Elemental composition of magnetic nanoparticles covered with polyacrylate.

	Atomic Concentration [%]	Mass Concentration [%]
Fe 2p	27.57	58.93
O 1s	50.99	31.22
C 1s	21.44	9.85

**Table 2:** Characteristics of C 1s and O 1s elements corresponding to high-resolution XPS spectra (* reference peak).

	Binding Energy [eV]	Relative Atomic Concentration [%]
C-C/C-H	* 285	64.54
C-O	286.5	9.32
C=O	288.5	26.14
O-Fe	530	61.26
O-C	531.3	33.26
O=C	533.1	5.48

**Table 3:** The values of the parameters of the magnetic field and concentration of magnetic nanoparticles, as well as the duration of application of the magnetic field used in superparamagnetic hyperthermia experiments.

No.	Magnetic Field Amplitude (G)	Magnetic Field Frequency (kHz)	Magnetic Nanoparticle Concentrations (mg/mL)	Duration of Application of Magnetic Field (min)
1	208	312.4	5	30
2	370	312.4	1	30

**Table 4:** Cell viability rate of the squamous cell skin carcinoma (A431) following the application of the magnetic field for duration of 30 min, a frequency (f) of 312.4 kHz, and different amplitudes (H), at 24 h post-exposure to magnetic field.

Magnetic Field Amplitude (G)		208(±1)	370(±1)
Cell viability (%) ± SD	Standard conditions (37 °C)	100 ± 1.03	100 ± 0.92
	Post-exposure to magnetic field	98.3 ± 1.44	86 ± 3.52

**Table 5:** Data of the cell viability percentages induced by magnetic suspensions under SPMHT on the human epidermoid squamous carcinoma (A431) cells. The results were normalized to control cells, considered 100(±0.71)%.

Sample/ Concentration	Cell Viability (%) ± SD A431 Cells after SPMHT (43(±0.1) °C for 30 min)	Cell Viability (%) ± SD A431 Control Cells
Fe_3_O_4_-PAA–(HP-γ-CDs)/ 1 mg/mL	54.04 ± 3.91	100 ± 0.71
Fe_3_O_4_-PAA–(HP-γ-CDs)/ 5 mg/mL	42.01 ± 2.68	
Fe_3_O_4_-PAA–(HP-γ-CDs)/ 10 mg/mL	17.2 ± 4.45	

**Table 6:** Recent quantitative data from magnetic hyperthermia with biocompatible nanoparticles (NPs) of Fe_3_O_4_ for skin cancer therapy (in vitro and in vivo) compared to our results.

NPs Type, Size	Biocompatibility, NP Concentration	Tumor Type	In Vitro (Cell Line)	In Vivo (Mouse Model)	Magnetic Field	Frequency	Therapy Temperature	Therapy Duration	Viability/Inhibition Cell/Tumor Volume	Ref.
Fe_3_O_4_; 8–12 nm	Coating with an amine-terminated silica shell of 6 nm, and functionalized with αvβ6 antibodies	Squamous carcinoma	VB6 cells line	-	9.7 mT	174 kHz	Hyperthermia temperature	10 min.	~50% viability at 24 h; ~25% viability at 48 h;	[35]
Fe_3_O_4_	CD44 antibody (CD44-Fe_3_O_4_: 100 nm); 0.4 mg/mL	Squamous carcinoma	-	BALB/c nude mice (male)	Current of 50 A in the induction coil	237 kHz	43 °C	30 min.	~33% inhibition	[36]
Fe_3_O_4_ in fibrous mat_._ (PCL); 50–100 nm	Polycaprolactone (PCL) fibrous bandage (10 mg)	Skin cancer	Cervical cancer HeLa cell lines (variant of Dox-resistant HeLa cells)	-	3.6 kA/m	236 kHz	45 °C	10 min.	~50% viability at 2 h	[37]
Fe_3_O_4_ in fibrous mat_._ (PCL); 50–100 nm	Polycaprolactone (PCL) fibrous bandage (10 mg)	Skin cancer	-	BALB/c mice (female)	3.6 kA/m	236 kHz	45 °C	15 min. according to therapy plan: 1z, 1z, 1z, 2z, 2z (z-day)	~7% decreases volume tumor at 24 h; ~82% decreases volume tumor at 2z; Complete tumor regression after 30 days of the treatment	[37]
Fe_3_O_4_; ~12 nm	1-PNP-hEGFR; 4.53 mg/mL	Squamous carcinoma	-	Epidermoid cancer in an A431 tumor-bearing mouse	25 kA/m	173 kHz	Hyperthermia temperature	30 min.	~60% decreases in tumor volume	[38]
Fe_3_O_4_; ~16 nm	Coated with PAA-(HP-γ-CDs); 10 mg/mL	Squamous carcinoma	A431 cells line	-	13.37 kA/m	312.4 kHz	43 °C	30 min.	17.2% viability at 24 h	This study

## Data Availability

Data are contained within article and Supplementary Materials.

## Supplementary Materials

The following supporting information can be downloaded at: https://www.mdpi.com/article/10.3390/ijms25158380/s1.
