# Supplementary material for: In Vitro Superparamagnetic Hyperthermia Employing Magnetite Gamma-Cyclodextrin Nanobioconjugates for Human Squamous Skin Carcinoma Therapy
Source: Int J Mol Sci. 2024 Jul 31;25(15):8380. doi: 10.3390/ijms25158380 (PMC11313510; doi:10.3390/ijms25158380)
Supplement: Supplementary file 1 [file ijms-25-08380-s001.zip › ijms-3073180-supplementary.pdf]

International Journal of
Molecular Sciences

Supplementary Material

In Vitro Superparamagnetic Hyperthermia Employing Magnetite Gamma-Cyclodextrin Nanobioconjugates for Human Squamous Skin Carcinoma Therapy

Isabela-Simona Caizer-Gaitan[1,2,3], Claudia-Geanina Watz[4,5], Costica Caizer[6,*], Cristina-Adriana Dehelean[5,7], Tiberiu Bratu[1], Zorin Crainiceanu[1], Adina Coroaba[8], Mariana Pinteala[8], and Codruta-Marinela Soica[5,9]

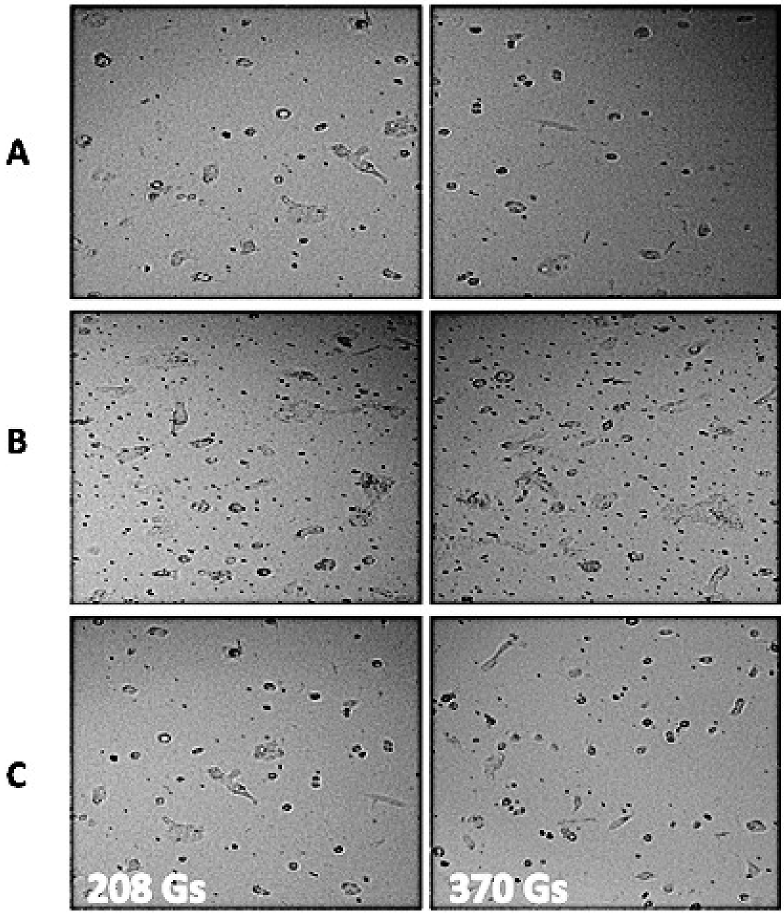

Figure S1. Cell density of squamous cell skin carcinoma - A431 cell line: (A) under standard conditions (37 °C), (B) laboratory conditions (24 °C), (C) after exposure to magnetic field for 30 minutes at frequency of 312.4(±0.01) kHz and amplitudes of 208(±1) and 370(±1) G. The images were captured at 24h post-exposure to experimental conditions, using 10X magnification.
